# THE RELATIONSHIP BETWEEN THE EXPERIENCE OF SIGNIFICANT HISTORICAL EVENTS AND COGNITIVE FUNCTION AMONG OLDER ADULTS

**DOI:** 10.1093/geroni/igz038.3406

**Published:** 2019-11-08

**Authors:** Stephanie M Bergren, Gabriella Dong

**Affiliations:** Rutgers, The State University of New Jersey, New Brunswick, New Jersey, United States

## Abstract

Research has found relationships between experiencing stressful events and lower cognitive function in late life. However, there is little research about the cumulative experiences of significant historical events and cognitive function. Historical events may be unique compared to other life events due to their potential distal relationship to the individual. This study aims to examine the relationship between experiencing significant historical events and cognitive function among Chinese older adults. Data were drawn from the PINE Study, a cohort study of 3,126 US Chinese older adults collected from 2017-2019. Participants were asked if they experienced the Japanese invasion, famine, Great Leap Forward, Vietnam War, Cultural Revolution, and the Tian’anmen Square Protests. A composite score of 0-6 was calculated to count the number experienced. Cognitive function was measured through global cognition, episodic memory, working memory, processing speed, and Chinese MMSE. Linear and quantile regression were performed. Among the participants, 1908 (61.04%) were female with mean age of 75.33 (SD=8.22) years. The average number of historical events experienced was 2.36 (SD=1.44). After adjusting for covariates, every one additional historical event experience was associated with better global cognition (b=0.26, SE=0.009, p<.01), episodic memory (b = 0.045, SE=0.012, p<.001), and processing speed (b=0.383, SE=0.135, p<.01). Number of historical events was not significantly associated with working memory or C-MMSE. The positive relationship between historical events and some cognitive domains suggests a potential resilience effect after experiencing historical events. Future research should examine whether participants found events stressful and whether there are differential relationships to cognitive function.

